# Assessment of late treatment-related symptoms using patient-reported outcomes and various factors affecting return to work in survivors of breast cancer

**DOI:** 10.3332/ecancer.2023.1533

**Published:** 2023-04-20

**Authors:** Rajeshwari Rai, Monica Malik, Deepthi Valiyaveettil, Syed Fayaz Ahmed, Mohammed Basalatullah

**Affiliations:** Nizam’s Institute of Medical Sciences, Punjagutta, Hyderabad 500082, India

**Keywords:** late symptoms, pre diagnosis, post treatment, return to work, household chores, employment, patient reported outcomes, fatigue, pain, social stigma

## Abstract

**Introduction:**

Breast cancer is the most common cancer in women worldwide. Survival in these patients has increased because of early diagnosis and multimodality treatment methods. Return to premorbid functional status after treatment is essential for rehabilitation and good quality of life. Many patients suffer from late treatment-related symptoms which affect their return to premorbid status. Various health-related and work-related variables also affect the return to premorbid status.

**Materials and methods:**

This is a cross-sectional study in which 98 patients with breast carcinoma who received curative treatment were included 6–12 months post-radiotherapy completion. Patients were interviewed to assess their type of work and hours of work prior to diagnosis and at the time of the study. The extent to which they are able to return to their pre-diagnosis level of work was noted and various factors that were hindering them were documented. Treatment-related symptoms were assessed using selected questions from NCI PRO-CTCAE (version 1.0) questionnaire.

**Results:**

The median age of diagnosis of patients included in the study was 49–50 years. The most common symptoms experienced by patients were fatigue (55%), pain (34%) and oedema (27%). 57% of patients were employed before diagnosis, of which only 20% were able to return to their employment post-treatment. All patients were involved in household work prior to diagnosis and 93% were able to get back to their routine household work, with 20% of patients requiring frequent work breaks. About 40% of patients reported social stigma as a factor that hindered them from returning to work.

**Conclusion:**

Most patients return to household work post-treatment. Fatigue, pain and social stigma were the most common barriers to return to employment. Patient-reported outcomes and functional assessments can enable better survivorship care.

## Introduction

Breast cancer is the most common cancer in women worldwide both in developed and developing countries. According to GLOBOCAN 2020, it is the leading cause of global cancer with an estimated 2.3 million new cases in 2020, representing 11.7% of all cases. Breast cancer is the fifth leading cause of cancer mortality (6.9%) [1]. There are about 1.7 million cases in India accounting for about 13.5% of all cancers and 10.6% of all deaths. Breast cancer accounts for about 25%–32% of all cancers in Indian women [2].

Standard of care is a multimodality approach which includes surgery (breast conservation surgery/modified radical mastectomy with or without axillary dissection), radiotherapy and systemic therapy (chemotherapy/endocrine therapy/targeted therapy). Survival in breast cancer patients has increased because of early diagnosis and multimodality treatment methods. According to the US statistics, the relative survival rate for women with breast cancer is 91% 5 years after diagnosis [3]. The 5-year survival rate for women in India is 66%, the causes of which are multifactorial including presentation in more advanced stages and limitations in access to care [4].

The most common late effects include pain, fatigue, lymphedema, peripheral neuropathy, loss of limb strength, and osteoporosis, all of which can affect patients’ participation in their day-to-day activities and employment [5].

Morbidity associated with the disease and its treatment (acute and late effects) leads to impairment in the physiological and psychological behaviours of patients, thus potentially limiting their ability to perform desired tasks [6].

Return to premorbid functional status after treatment is essential with regard to rehabilitation and quality of life. Various health-related and work-related variables influence the return to premorbid functional status in these women. Factors such as support from family, working environment and employer support also affect the return to work (RTW) [7].

In India, the diagnosis of cancer is associated with cultural and social stigma, familial distancing and social isolation [8]. Self-stigma due to treatment effects (lymphedema, weight gain, hair loss and loss of breast) has also been observed in these women. This can affect the quality of life [9].

In India, women in the low and middle socio-economic status groups are mostly involved in daily labour work or household work. There is very little evidence on survivorship issues in Indian women treated for breast cancer. There are few studies on factors influencing RTW among breast cancer survivors in India. Current care for these survivors on follow-up focuses on the detection and treatment of recurrence, with little or no attention to the physical, psychosocial and functional well-being of the patient [5].

Symptoms experienced by breast cancer survivors are under-reported. This can lead to a negative impact on their daily function. Evaluation of post-treatment symptoms using patient-reported outcomes can be useful to address the symptoms and provide proper rehabilitation [10].

Our study aimed to assess the late treatment-related symptoms using patient-reported outcomes and various factors that affect RTW in survivors of breast cancer.

## Methods

A cross-sectional study was conducted on 100 patients of localised breast cancer who have received curative treatment and are on follow-up for at least 6–12 months after completing radiotherapy. Socio-demographic details, disease and treatment-related details were collected from case records.

Patients were interviewed to assess their type of work and hours of work prior to diagnosis and at the time of the study. The extent to which they are able to return to their pre-diagnosis level of work was noted and various factors that were hindering them were documented.

Return to household chores and return to employment were assessed separately and quantified in terms of full/partial/none depending on the number of hours spent in household chores/employment.

Treatment-related symptoms and various factors hindering them were assessed using selected questions from **NCI PROCTCAE** (version 1.0) questionnaire. Selected post-treatment symptoms commonly experienced in breast cancer patients (6 symptoms – arm swelling, tingling and numbness, insomnia, fatigue, pain and anxiety) were included in the questionnaire (15 questions).

## Results

**Figure 1.**
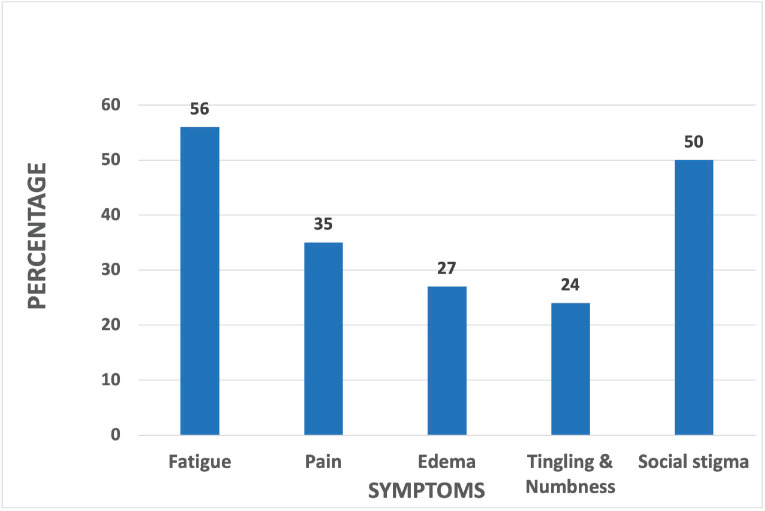
Late treatment related symptoms.

Around 98 patients were recruited in the study. Their disease, treatment and pre-diagnostic work-related details are mentioned in Table 1. Most commonly reported late treatment-related symptoms were fatigue – 56% (*n* = 55), pain – 35% (*n* = 34), oedema – 27% (*n* = 27) and tingling and numbness – 24% (*n* = 24). Social stigma was reported in 50% (*n* = 49) of patients (Figure 1).

### Return to household work

All the women were engaged in household work prior to diagnosis of cancer. 90 out of 98 (92%) of them were able to get back to their household chores post-treatment. 20 of 90 (22%) women, who were able to carry out their regular household chores, required frequent breaks in carrying out their daily tasks. The most commonly experienced symptoms that hindered these 20 women from carrying out their work without breaks were – fatigue in 18 and pain in 7 women. 55% (*n* = 11/20) reported social stigma as a factor affecting their complete return.

8 out of 98 (8%) women were not able to return to their regular household chores. The most common factors hindering their return were age >45 years and low education status. The most common symptoms reported by these patients were – pain in five women while three women had fatigue, arm oedema, tingling and numbness, and insomnia. Four of these women reported social stigma as a hindering factor.

### Return to employment

54 out of 98 (55%) women were employed prior to diagnosis. 19 out of these 54 (35%) were able to get back to their employment post-treatment and the rest 65% (*n* = 35) were unable to get back to employment. The most common associated factors hindering them from RTW were age>45 years in 54% (19/35), low education status in 68% (*n* = 24/35) and involvement in manual physical labour in 83% (29/35). The most commonly reported symptoms were – fatigue in 60% (21/35) and pain in 57% (20/35). 72% (25/35) of women reported social stigma as a hindering factor.

## Discussion

There are sparse data on RTW in survivors of breast cancer from developing countries. Household chores constitute a significant amount of workload for many women. Most studies report on return to employment and ignore the burden of household work. Social and cultural factors also necessitate that women are actively engaged in household duties. This study attempted to focus on both these aspects of work in women from low and middle socioeconomic status. Survivorship care is predominantly focused on the assessment of disease status. Quality of life issues including RTW are often not addressed and no specialised services are available in this regard.

In our study, the majority of the survivors were able to carry out their regular household chores; however, 22% of them required frequent breaks in carrying out their daily household work. Nearly two-thirds of those who were employed prior to diagnosis were not able to return to their employment. These results were found to be consistent with previous studies conducted across the world, which have reported poorer quality of life and decreased RTW in survivors of breast cancer following the treatment. Multiple factors are associated with the probability of RTW. Many studies [11, 12] have reported that younger age, higher education level, single status, high income and good employer support are associated with higher levels of RTW. On the other hand, old age, low education level, low income and poor support were barriers to RTW.

Evidence suggests that women prioritise returning to productive activities including household work rather than to social activities [13]. Disease and treatment-related symptoms including cognitive effects are major barriers to RTW, emphasising the importance of addressing these issues during follow-up care. Patient-reported outcomes can be helpful in effectively documenting post-treatment symptom burden towards a more effective resolution. Work environment including support from employers and colleagues may significantly facilitate RTW.

Studies also reveal a significant association of physical, cognitive and psychological symptoms with impaired RTW and reduced level of work in a significant proportion of survivors of breast cancer [14]. Various studies [9, 12, 15, 16] showed that women involved in physical work had decreased rates of RTW and difficulties adjusting to their work compared to non-manual workers. A systematic review of 26 studies [16] reported that sociodemographic factors like education and ethnicity, treatment-related factors such as side effects of chemotherapy, work-related factors like heavy physical work and disease-related factors like depression and emotional distress act as barriers to RTW in breast cancer survivors. Social, family and employer support, and financial independence facilitate RTW.

Survivors often report chronic pain in the cervical spine and axilla. Unrelieved pain is often associated with lower rates of RTW [17]. Pain management and vocational rehabilitation should be incorporated into survivorship care to improve quality of life.

Many studies have reported a significant prevalence of social stigma in breast cancer survivors [9, 18, 19]. This may vary among different countries depending on socio-cultural environments. There is, however, a dearth of literature on the association of stigma with RTW. Stigma was reported by a significant proportion of survivors in our study as a hindering factor for RTW including domestic work and employment. Stigma can be multifactorial including issues with body image and negative public attitudes. Higher levels of stigma have been reported in women with low education status, young age, and in those who underwent mastectomy compared to breast conservation surgery [20]. Self-perceived and social stigma, physical factors (fatigue, pain and lymphedema) and psychological factors have been shown to impact the ability to return to everyday life activities and work [9]. A multi-pronged approach is needed to analyse and address factors related to self-perceived and social stigma in cancer survivors.

Factors related to RTW are understudied, especially in LMICs, and are poorly documented or addressed in survivorship care. A large proportion of survivors struggle with physical, cognitive and psychosocial issues which affect their social and vocational rehabilitation. A comprehensive and multidimensional approach is needed to effectively address these issues and improve the overall quality of life in these women.

Limitations of our study include small sample size and short follow-up. Longer post-treatment follow-ups may be associated with a larger proportion of RTW. We did not conduct an in-depth analysis of the associated factors including stigma.

## Conclusion

In this study, we found a significant proportion of patients were not able to return to employment. A high prevalence of fatigue, pain and stigma was found in patient-reported outcomes. RTW requires appropriate multidisciplinary interventions in order to provide a satisfactory return to normal life.

Further studies with a larger number of survivors and longer follow-ups are needed to substantiate these findings. There is also a need for a proper design of an interventional model to identify and address these factors towards improving the overall quality of life.

## Funding

None.

## Conflicts of interest

None.

## Figures and Tables

**Table 1.** Patient characteristics.

Age Less than 45 years More than 45 years	40% (n = 39) 60% (n = 59)
Educational status Illiterate Educated	55% (*n* = 54) 45% (*n* = 44)
Type of work Household work Employment	100% (*n* = 98) 55% (*n* = 54)
Intensity of work Manual physical work Sedentary work	36% (*n* = 35) 64% (*n* = 63)
Comorbidities Yes No	46% (*n* = 45) 54% (*n* = 53)
Site of cancer Right side Left side Bilateral	62% (*n* = 61) 36% (*n* = 36) 2% (*n* = 1)
Stage of tumour Tis IA IIA IIB IIIA IIIB IIIC	2% (*n* = 2) 3% (*n* = 3) 28.5% (*n* = 28) 22.4% (*n* = 22) 19.4% (*n* = 19) 13.26% (*n* = 13) 11.22% (*n* = 11)
Surgery Modified radical mastectomy Breast conservation surgery No surgery	70% (*n* = 69) 28% (*n* = 27) 2% (*n* = 2)
Chemotherapy Yes No	93% (*n* = 91) 7% (*n* = 7)
Hormone receptors Positive Negative Her2neu Positive Negative	47% (*n* = 46) 53% (*n* = 52) 33% (*n* = 32) 67% (*n* = 66)
Radiotherapy Yes	100%
